# Notes on Preparations for the Sick

**Published:** 1909-06

**Authors:** 


### Notes on Preparations for the Sick.

**"Diamalt."**—THE BRITISH DIAMALT CO.—This preparation is described as a pure and highly diastasic extract of malt. We have examined its digestive action on starch at the temperature of the body, and found it to be quite satisfactory.

[A digestive experiment was made in an incubator just below 40° C. During the night a considerable amount of starch was converted into sugar.—O.C.M.D.]

---

**"Glidine."**—MENLEY AND JAMES LTD.—Under the above name a food preparation has been placed on the market, and the proprietors claim that it is a purely vegetable protein food, prepared wholly from wheat. It is also stated to contain lecithin, and to be practically free from carbohydrates. So many foods are described as free from carbohydrates that it was deemed desirable to make a fairly complete analysis. A microscopic examination revealed a vegetable structure, but starch grains were almost entirely absent. We have never examined such a preparation containing less. No sugar could be detected. An attempt was made to extract lecithin, but it was not found possible to obtain a sufficient quantity for identification. As an unsweetened food, rich in proteids and exceptionally free from carbohydrates, we can strongly commend "Glidine," especially if prepared with milk, so that additional fat may be introduced.

ANALYSIS OF "GLIDINE" (by O.C.M.D.).

| | | |
|---|---|---|
| Moisture .. .. .. .. .. .. | 9·20 % | (9·20) |
| Fat .. .. .. .. .. .. .. | 0·42 % | (0·42) |
| Ash .. .. .. .. .. .. .. | 0·44 % | (0·44) |
| Proteids .. .. .. .. .. .. | 89·50 % | (89·50) |
| Cellulose, etc. .. .. .. | 0·44 % | (0·44) |
| | 100.00 | (100.00) |

**Iodoglidine.**—MENLEY AND JAMES LTD.—An organic compound of iodine with protein. The separation of the iodine from the albumin takes place almost wholly in the intestine, only traces of iodine being liberated in the stomach. The iodine is gradually and slowly liberated and slowly absorbed. It therefore gives the fullest benefit, and toxic symptoms are not likely to arise.

